# The emerging role of cuproptosis in spinal cord injury

**DOI:** 10.3389/fimmu.2025.1595852

**Published:** 2025-06-16

**Authors:** Daoran Xu, Liyu Hu, Jinming Zhou, Xiongwei Deng, Yunrong Zhu, Chao Liu

**Affiliations:** Department of Spinal Surgery, Affiliated Jiangyin Clinical College of Xuzhou Medical University, Jiangyin, China

**Keywords:** spinal cord injury, reactive oxygen species, copper homeostasis, cuproptosis, programmed cell death

## Abstract

Copper is a vital trace element integral to numerous biological processes, including iron metabolism, neurotransmitter synthesis, mitochondrial respiration, oxidative stress regulation, and energy production. However, disturbances in copper metabolism can result in pathological conditions, including cuproptosis—a newly recognized form of programmed cell death (PCD) marked by copper accumulation and the disruption of copper-dependent metabolic pathways. Cuproptosis has been associated with various diseases, such as cancer, metabolic disorders and neurodegenerative disorders. In the context of spinal cord injury (SCI), multiple pathological mechanisms, including oxidative stress, inflammation, and PCD could impact the patient’s prognosis with SCI. This review seeks to elucidate the pathophysiological underpinnings of SCI, the mechanisms and biological significance of copper homeostasis and the role of cuproptosis in SCI.

## 1 Introduction

Copper, an essential transition metal, serves as a critical cofactor for fundamental biological processes ranging from mitochondrial electron transport to neurotransmitter biosynthesis (1–3). While the human body maintains copper concentrations within a narrow physiological range (~100–200 mg total), this delicate homeostasis represents a metabolic tightrope walk. Both copper deficiency and overload trigger severe pathologies—from Menkes disease (MD)/Wilson disease (WD) to cancer progression—highlighting the metal’s Janus-faced role in human health (4, 5). The recent discovery of cuproptosis, a copper-dependent cell death modality driven by mitochondrial copper accumulation, has reshaped our understanding of copper’s pathophysiological roles. Unlike apoptosis or ferroptosis, cuproptosis uniquely involves Ferredoxin 1 (FDX1)-mediated lipoylated protein aggregation and iron-sulfur (Fe-S) cluster disassembly, establishing it as a mechanistically distinct player in disease pathogenesis (6).

**Figure 1:**
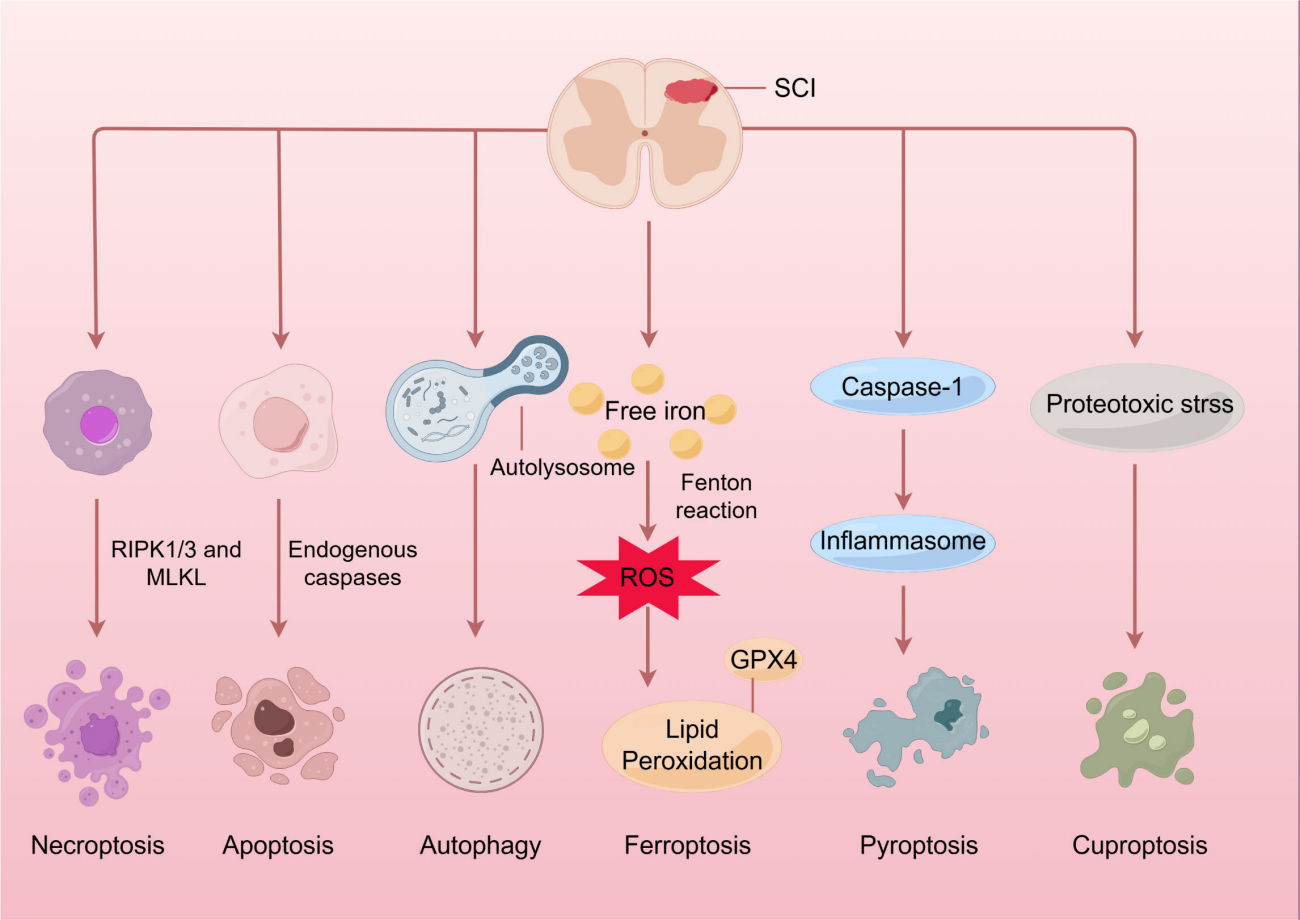
Spinal cord injury and programmed cell death. Initially, the application of mechanical force damages the cell membrane and releases intracellular components, further triggering autophagic processes as a defense mechanism. Following this, a range of pathophysiological processes occur, including oxidative stress, inflammation, and immune response, which eventually result in the apoptosis, necroptosis, and pyroptosis of neuronal and glial cells through specific mechanisms. Moreover, an overabundance of ROS may lead to lipid peroxidation and trigger ferroptosis. It’s intriguing that an excess of copper at the injury site might induce mitochondrial proteotoxic stress and cause cuproptosis. SCI, Spinal cord injury; RIPK1/3, receptor-interacting protein kinases; MLKL, mixed lineage kinase domain-like protein; ROS, reactive oxygen species; GPX4, glutathione peroxidase 4.

Emerging evidence implicates cuproptosis in neurodegenerative disorders and malignancies (7–9), with newly published studies elucidating its potential role in spinal cord injury (SCI) progression (10, 11). SCI is a prevalent and catastrophic form of central nervous system (CNS) injury that results in motor, sensory, and autonomic dysfunction. As of 2016, there were approximately 27 million cases globally (12). Furthermore, SCI imposes a substantial medical burden, with annual medical expenditures for SCI patients in the United States reaching $3 billion in 2021 (13). In the context of SCI, cuproptosis may contribute to proteotoxic stress, mitochondrial dysfunction, and inflammatory responses, thereby exacerbating tissue damage (14–16). SCI is a complex condition characterized by both primary and secondary injury mechanisms (17). The primary injury is generally induced by a mechanical force that disrupts spinal cord tissue, resulting in immediate cell death and the loss of axonal connectivity (18). Subsequently, a series of secondary injury processes ensue, including oxidative stress, inflammation, excitotoxicity, and programmed cell death (PCD), which further exacerbate the initial damage and promote additional tissue degeneration (13, 18–20) (**Figure 1**).

Research by Salsabili et al. has demonstrated that serum copper concentrations are significantly elevated in patients with spinal cord injuries compared to healthy individuals (21). The accumulation of copper in SCI initiates an inflammatory response, marked by the activation of microglia, which subsequently leads to the release of pro-inflammatory cytokines and chemokines. This inflammatory cascade further exacerbates tissue damage and impedes recovery (22, 23). Elucidating the role of cuproptosis in SCI is crucial for the development of targeted therapeutic interventions. Potential therapeutic strategies may involve the use of copper chelators to reduce copper accumulation and agents that modulate cuproptosis and mitochondrial function (12, 14, 24). Comprehensive therapeutic strategies that address multiple cellular pathways are imperative.

This review seeks to elucidate the mechanisms and biological functions of cuproptosis, the pathophysiological processes underlying SCI, the role of cell death in SCI, and the emerging significance of cuproptosis in the context of SCI. By comprehending the interplay between cuproptosis and SCI, novel therapeutic strategies can be developed to mitigate cell death and promote spinal cord repair. Further research is imperative to clarify the precise mechanisms and therapeutic potential of targeting cuproptosis in SCI.

## 2 Pathophysiological mechanisms of spinal cord injury

SCI represents a devastating traumatic insult to the CNS, inducing sensorimotor deficits and autonomic dysregulation that irrevocably compromise patients’ quality of life while imposing staggering socioeconomic burdens (12, 25). The prevailing pathophysiological paradigms underlying SCI are intricate and multidimensional, encompassing both primary and secondary injury processes (17). A comprehensive understanding of the interactions among these mechanisms is crucial for the development of effective therapeutic interventions.

### 2.1 Primary injury mechanisms

SCI predominantly results from acute mechanical forces that damage the integrity of spinal cord tissue, inducing structural compromise through compression, distraction, or shearing mechanisms (26). The biomechanical insult triggers a series of pathological processes: on the one hand, the mechanical force causes significant disruption to neuronal and glial cells, resulting in immediate cell death and the loss of axonal connectivity (27, 28). On the other hand, the Blood-Spinal Cord Barrier (BSCB), which ordinarily serves to protect the spinal cord from potentially harmful substances in the bloodstream, becomes compromised, allowing the infiltration of these substances (29). Additionally, the injury triggers the release of pro-inflammatory cytokines and chemokines, setting the stage for the secondary injury cascade (30, 31).

### 2.2 Secondary injury mechanisms

#### 2.2.1 Oxidative stress

Oxidative stress constitutes a principal secondary injury mechanism in SCI. Subsequent to the initial mechanical trauma, a cascade of progressive processes is initiated, culminating in additional neuronal damage and functional impairment (12, 18). Oxidative stress response is mainly characterized by high levels reactive oxygen species (ROS), including superoxide anion radical (O_2_-), hydrogen peroxide (H_2_O_2_), hydroxyl radical (·OH), and peroxynitrite (32). It can be seen as a double-edged sword. Initially, low oxidative stress levels might help protect damaged tissues and cells (33). Yet, prolonged high oxidative stress and excessive ROS production can worsen further the inflammatory response and trigger various PCD pathways (34, 35). Mitochondria, which are not only essential for cellular energy metabolism and mitochondrial homeostasis, but also are the main organelles that generate ROS after SCI, play a significant role. This secondary injury is marked by an elevated production of ROS, which can surpass the cellular antioxidant defenses, thereby inducing mitochondrial dysfunction and apoptosis (36, 37).

Within the pathomechanical cascade of SCI, the disruption of mitochondrial homeostasis is of paramount importance, since mitochondria are central to energy production and cell death regulation (14, 38). The accumulation of oxidative stress not only facilitates neuronal apoptosis but also intensifies inflammation and tissue damage (39). Empirical studies have demonstrated that interventions designed to mitigate oxidative stress can substantially enhance outcomes in SCI models, underscoring the significance of targeting this mechanism in therapeutic strategies (34, 40, 41).

Furthermore, the interaction between oxidative stress and inflammatory responses adds complexity to the injury process. Consequently, comprehending the role of oxidative stress in SCI is essential for devising effective strategies to alleviate secondary injury and facilitate recovery. Therapeutic strategies aimed at mitigating oxidative stress and its downstream effects may offer potential for enhancing functional recovery and outcomes in patients with spinal cord injuries (12, 18).

#### 2.2.2 Inflammation

Inflammation constitutes a pivotal element of the secondary injury cascade. Subsequent to the initial mechanical trauma, a multifaceted series of inflammatory responses is activated, potentially exacerbating damage and impeding recovery (42). While the primary injury results in immediate cellular damage, it is the ensuing inflammatory processes that substantially contribute to the magnitude of secondary injury (23). This cascade is initiated within hours post-trauma and may persist for several months, engaging various immune cells and signaling molecules that can either facilitate healing or precipitate further degeneration (18, 42, 43).

The inflammatory response is typified by the infiltration of immune cells, including neutrophils and macrophages, which secrete pro-inflammatory cytokines and chemokines (40, 44). These signaling molecules, such as IL-1β, TNF-α, and IL-6, are crucial in orchestrating the inflammatory response and may directly result in heightened oxidative stress and apoptosis of functional cells, such as neurons (45, 46). Besides, the chemokine CCL3, which is instrumental in the recruitment of inflammatory cells, has been demonstrated to exacerbate secondary damage following SCI by promoting inflammation and enlarging the lesion area (47). Similarly, C-C motif chemokine ligand 20 (CCL20) has been implicated in regulating neuroinflammation through the recruitment of Th17 cells, further complicating the inflammatory landscape following SCI (48).

In summary, inflammation should not be regarded solely as a byproduct of SCI; rather, it plays a pivotal role in the secondary injury cascade. The regulation of inflammation is essential for mitigating damage and facilitating recovery, underscoring the necessity for targeted therapeutic interventions that can effectively modulate the inflammatory response (23, 49).

#### 2.2.3 Excitotoxicity

Excitotoxicity is a pathological process resulting from the excessive activation of excitatory neurotransmitters, notably glutamate (50). In the pathomechanistic framework of SCI, the initial trauma triggers a series of biochemical events that intensify neuronal damage, predominantly through the excessive release of glutamate (51). This surplus of glutamate can induce neuronal death and additional complications by activating various receptors, including NMDA receptors. While NMDA receptors play a vital role in synaptic plasticity, their overstimulation can lead to excitotoxicity (52, 53).

Within the process of SCI, neurotrauma causes a rapid increase in extracellular glutamate, which results in excitotoxicity, inflammation, glial scar development, and ultimately neuronal death. This elevation is contingent upon the severity of the injury, with more severe injuries correlating with higher levels of excitatory amino acids (51, 54). Furthermore, excitotoxicity not only facilitates immediate neuronal death but also predisposes the tissue to secondary injury mechanisms, including mitochondrial dysfunction and persistent neuroinflammation (55, 56).

Research indicates that interventions designed to modulate glutamate levels can effectively mitigate the effects of excitotoxicity. For example, the administration of pharmacological agents such as levetiracetam has shown potential in facilitating functional recovery following SCI by stabilizing astrocytes, which are essential for glutamate uptake and homeostasis (57). The removal of CNS glutamate into the systemic blood circulation through intravenous (IV) administration of blood glutamate scavengers (BGS) such as the enzyme recombinant glutamate-oxaloacetate transaminase 1 (rGOT1) and its co-substrate. The availability of the methods to remove excess glutamate from CNS without crossing the blood-brain barrier and with little to no negative effects could offer a substantial therapeutic benefit (54). Interestingly, mitochondria both aggravate glutamate toxicity due to impairments in the tricarboxylic acid (TCA) cycle and become a victims of glutamate toxicity, which disrupts oxidative phosphorylation (58).

#### 2.2.4 Types of cell death in spinal cord injury

##### 2.2.4.1 Apoptosis

Apoptosis is a key pathological feature of secondary cord injury, resulting in spinal cord dysfunction and permanent irreversible changes in neuronal cells, including neurons, astrocytes, oligodendrocytes, and microglia (59, 60). Caspases plays a crucial role in mediating apoptosis and is key to controlling this process (61). To date, there are three principal signaling pathways implicated in apoptosis encompassing the intrinsic (mitochondrial), extrinsic (death receptor), and endoplasmic reticulum (ER) pathways (61, 62). Within the context of SCI, apoptotic pathways of these damaged cells is predominantly initiated by factors such as oxidative stress, inflammation, and excitotoxicity (63). Oxidative stress, resulting from mitochondrial dysfunction and the generation of ROS, culminates in cellular damage and the activation of the intrinsic apoptotic pathway (64). Inflammation, marked by the secretion of pro-inflammatory cytokines such as tumor necrosis factor-alpha (TNF-α) and interleukin-1 beta (IL-1β), initiates the extrinsic pathway of apoptosis (45, 65). Excitotoxicity, induced by the excessive activation of excitatory neurotransmitters such as glutamate, leads to calcium influx and subsequent cellular damage, thereby further promoting apoptotic processes (14, 51).

The intrinsic apoptotic pathway is initiated by cellular stress, which prompts the release of pro-apoptotic proteins (cytochrome c) into the cytoplasm by increasing the permeability of mitochondrial membrane. This event facilitates the formation of caspase-3 and caspase-7 by activating caspase-9 and Cyt-c, subsequently activating apoptosis and regulating other endogenous apoptotic cascades leading to cell death (66). In contrast, the extrinsic apoptotic pathway is triggered by the interaction of death ligands, such as Fas ligand (FasL) with their corresponding death Fas receptors. This interaction results in the recruitment of adaptor proteins, such as Fas-associated death domain protein (FADD), consequently activating pro-caspase-8 and forms a death-inducing signaling complex (DISC), thereby mediating the executive stage of apoptosis (61, 65, 67). ER stress arises from the accumulation of misfolded proteins within the ER, which activates the unfolded protein response (UPR), increasing the expression of apoptotic proteins, and leading to apoptosis in spinal cord neurons (62).

In the pursuit of effective therapeutic interventions to mitigate apoptosis in SCI, several promising strategies have emerged. Caspase inhibitors, such as zVAD-fmk, function by obstructing the activation of caspases, thereby preventing apoptosis (68). The administration of ligustilide (LIG) post-SCI enhances mitophagy through the BNIP3-LC3 interaction, reduces oxidative stress, restores normal mitochondrial function, and ultimately weakens the intrinsic pathway of apoptosis (69). Zhou et al. have confirmed that stem cells and exosomes prevent apoptosis following SCI by modulating Fas/FasL-mediated extrinsic pathways (70). Furthermore, in SCI model rats, nerve growth factor (NGF) has been demonstrated to reduce neuronal apoptosis and suppress the ER stress-induced apoptotic response proteins CHOP, GRP78, and caspase-12 (71). A comprehensive understanding of the initiating factors, key signaling pathways, and potential therapeutic strategies to attenuate apoptosis is crucial for advancing treatment approaches. However, further research is essential to substantiate these strategies and to formulate comprehensive treatment protocols for SCI.

##### 2.2.4.2 Autophagy

Autophagy is a crucial cellular process that relies on lysosomes to degrade damaged organelles, misfolded proteins and other cellular materials for recycling and clearing, thereby mitigating cellular toxicity (72). There are currently three well-known types of autophagy: macroautophagy, microautophagy, and chaperone-mediated autophagy (CMA). Generally, the term autophagy refers to macroautophagy (73). Autophagy in SCI is predominantly initiated by a variety of factors, including oxidative stress, nutrient deprivation, and mechanical injury. Oxidative stress, which arises from mitochondrial dysfunction and the subsequent generation of ROS, results in cellular damage and triggers autophagy as a protective mechanism (12). Similarly, nutrient deprivation, frequently observed following injury, activates autophagy to preserve cellular homeostasis (74). Excessive autophagy, however, can lead to the autophagic death of spinal cord neurons, exacerbating tissue damage. Hence, tight regulation of autophagy is essential to maintain optimal levels (13, 75). Besides, mechanical injury associated with SCI can lead to damage of the cell membrane and the release of intracellular components, thereby further stimulating autophagic processes (76).

Key signaling pathways involved in the regulation of autophagy encompass the mTOR pathway, the AMPK pathway, and the ULK1 complex (76). The mTOR pathway serves as a principal regulator of autophagy; specifically, the inhibition of mTOR is associated with the promotion of autophagic processes (77). Conversely, the AMPK pathway is activated in response to cellular energy stress, resulting in the inhibition of mTOR and the subsequent activation of autophagy (78). The ULK1 complex, which comprises ULK1, ATG13, FIP200, and ATG101, plays a critical role in the initiation of autophagy (79). Recent studies have further elucidated mechanisms of autophagy following SCI, particularly emphasizing the importance of mitophagy (12, 80). Mitophagy, a specialized form of autophagy targeting damaged mitochondria, is activated in response to mitochondrial damage, oxidative stress, and ROS (81). This process is crucial for the clearance of damaged mitochondria and the maintenance of cellular homeostasis.

Potential therapeutic strategies for modulating autophagy in SCI include the use of mTOR inhibitors, AMPK activators, and autophagy agonists. mTOR inhibitors, such as rapamycin and KU0063794, have been demonstrated to enhance autophagy, thereby reducing neural tissue damage and locomotor impairment following SCI (82, 83). Similarly, AMPK activators, including Netrin-1, promote autophagic processes by inhibiting mTOR, leading to improved functional recovery in rat models of SCI (84). Additionally, autophagy agonists like resveratrol have also been shown to facilitate functional recovery and reduce neuroinflammation through the activation of autophagy via the AMPK/mTOR pathway post-SCI (78).

In conclusion, autophagy plays a crucial role in the pathophysiology of SCI, supporting cellular protection and recovery processes (13). Targeting autophagy may provide a viable approach to alleviating secondary injury and enhancing functional recovery in individuals with SCI (76). Continued research is imperative to substantiate these approaches and to formulate comprehensive treatment protocols for SCI.

##### 2.2.4.3 Pyroptosis

Pyroptosis, identified as a PCD in 2001, primarily takes place in macrophages, dendritic cells, and neutrophils, but can also be seen in neuron cells (85, 86). It is a lytic, pro−inflammatory PCD program and characterized by its reliance on gasdermin proteins as the executioners of cell death (86). Pyroptosis in SCI is predominantly initiated by various factors, including infection, immune activation, and mechanical trauma. Infections, especially those caused by pathogens that stimulate the inflammasome, result in the production of pro-inflammatory cytokines and the subsequent induction of pyroptosis (87). Immune activation, marked by the release of danger-associated molecular patterns (DAMPs) and pathogen-associated molecular patterns (PAMPs), also triggers inflammasome activation and facilitates pyroptosis (88, 89). Mechanical trauma, exemplified by SCI, can result in damage to the cell membrane and the subsequent release of intracellular components, thereby triggering inflammasome activation (87).

Critical signaling pathways implicated in pyroptosis encompass the NLRP3 inflammasome, AIM2 inflammasome, and caspase-4/5/11 pathways (86). The NLRP3 inflammasome is triggered by diverse stimuli, such as ROS formation, lysosomal rupture, and ion channel gating (90).This activation triggers the formation of the inflammasome complex and then activates caspase-1. Once activated, caspase-1 cleaves pro-interleukin-1β (pro-IL-1β) and pro-interleukin-18 (pro-IL-18) into their active forms, which are subsequently released into the extracellular space, thereby promoting inflammatory responses (91, 92). Moreover, caspase-1 cleaves gasdermin D, resulting in the formation of pores in the cell membrane and subsequent cell lysis (93). The AIM2 inflammasome is induced by the presence of cytosolic DNA, which triggers the assembly of the inflammasome complex and the activation of caspase-1 (87, 94). The caspase-4/5/11 pathways are initiated by bacterial lipopolysaccharide (LPS) and directly cleave gasdermin D, resulting in pyroptosis (95).

Potential therapeutic strategies for mitigating pyroptosis in SCI include the use of inflammasome inhibitors, anti-inflammatory agents, and autophagy modulators. Inflammasome inhibitors such as MCC950 and Topotecan (TPT) have demonstrated efficacy in inhibiting the activation of the NLRP3 inflammasome, thereby reducing pyroptosis and neuronal death, and enhancing functional recovery post-SCI (96, 97). Anti-inflammatory agents, such as inhibitors targeting IL-1β and IL-18, have been shown to decrease the release of pro-inflammatory cytokines and attenuate pyroptosis (98, 99). Notably, Betulinic acid (BA), an autophagy modulator, has been shown to regulate autophagy and mitophagy through the AMPK-mTOR-TFEB signaling pathway, significantly inhibiting pyroptosis and facilitating functional recovery following SCI (100). By focusing on the modulation of pyroptosis, it may be possible to mitigate secondary injury and enhance functional recovery in patients with SCI.

##### 2.2.4.4 Necroptosis

Necroptosis refers to one form of PCD mediated by genetic programming and regulatory processes (101). Necroptosis is triggered by interactions involving death receptor family ligands with agonists such as tumor necrosis factor (TNF), FasL, and TRAIL (102). Current studies have shown that necroptosis plays a crucial role in the pathogenesis of SCI. This regulated necrotic process, orchestrated by receptor-interacting protein kinases (RIPK1 and RIPK3) and the mixed lineage kinase domain-like protein (MLKL) family, significantly influences the inflammatory response and neuronal and glial cell death subsequent to SCI (103–105).

After SCI, the accumulation of RIPK1 and RIPK3 proteins is associated with lysosomal dysfunction and the suppression of autophagy, both of which are vital for maintaining cellular homeostasis and survival. Following rapamycin treatment, lysosomal function is restored in cells, resulting in decreased levels of RIPK1, necroptosis, and cell death (103). The interaction between necroptosis and neuroinflammation is especially significant, as the inflammatory milieu resulting from necroptotic cell death can exacerbate neuronal damage and impede recovery (106, 107).

Inhibitors of RIPK1, such as necrostatins, have demonstrated potential in preclinical models by decreasing cell death and enhancing functional outcomes (108). MLKL is a pivotal regulator of necroptosis. MLKL levels increased significantly post-SCI, and inhibition of MLKL improved recovery of neurological function in SCI (105). Elucidating the mechanisms underlying necroptosis in SCI not only advances our understanding of the injury process but also facilitates the development of novel interventions aimed at promoting recovery and regeneration in the injured spinal cord (13, 102).

##### 2.2.4.5 Ferroptosis

Ferroptosis, a well-known PCD and first formally defined in 2012, is characterized by disturbances in iron metabolism and the accumulation of lipid reactive oxygen species (109). In SCI, ferroptosis is predominantly initiated by factors such as oxidative stress, iron overload, and the dysregulation of lipid metabolism. Oxidative stress, resulting from impaired mitochondrial function and the generation of ROS, contributes to lipid peroxidation and the subsequent activation of ferroptosis (12). Additionally, iron overload could further intensify lipid peroxidation and the ferroptotic process (110). The dysregulation of lipid metabolism, characterized by the accumulation of polyunsaturated fatty acids (PUFAs) and the deficiency in various elements of the antioxidant glutathione (GSH) pathway, significantly contributes to the process of ferroptosis (111, 112).

Central to the signaling pathways implicated in ferroptosis are the GPX4-dependent and ACSL4-dependent pathways. The enzyme glutathione-dependent antioxidant enzyme glutathione peroxidase 4 (GPX4) is instrumental in safeguarding cells against ferroptosis by reducing lipid peroxides. Inhibition or deficiency of GPX4 results in the accumulation of lipid peroxides, thereby triggering ferroptosis (111, 113). Conversely, the enzyme Acyl-CoA synthetase long-chain family member4 (ACSL4) facilitates the synthesis of lipid substrates necessary for lipid peroxidation, thereby promoting ferroptosis (114). Recent studies have elucidated further mechanisms involved in ferroptosis within SCI, highlighting the significance of mitochondrial quality control (MQC). Mitochondria play a crucial role in modulating ferroptosis by influencing iron homeostasis, energy metabolism, and lipid synthesis (115, 116).

Several studies have demonstrated that ferroptosis inhibitors, such as ferrostatin-1 and Celastrol, can reduce ROS accumulation, thereby inhibiting ferroptosis in oligodendrocytes and ultimately promoting functional recovery following SCI (117, 118). Alpha-tocopherol (Vitamin E, Vit E), an effective antioxidant, has been shown to mitigate ROS accumulation, iron overload, lipid peroxidation, and mitochondrial dysfunction, thereby alleviating SCI-induced ferroptosis by downregulating Alox15 in rats with SCI (119). Interestingly, there is a notable rise in iron accumulation in the motor cortex of both SCI patients and rats, triggering the accumulation of lipid ROS and causing motor neuron ferroptosis (120). Iron deposition is a critical pathological event in ferroptosis; deferoxamine (DFO), a potent iron chelator, has been shown to increase the expression levels of GPX4, xCT, and GSH, thereby improving survival of neuronal survival and inhibiting gliosis (121). To conclude, targeting ferroptosis may offer a promising way to reduce secondary injury and improve functional recovery by these potential therapeutic strategies in patients with SCI.

## 3 Molecular mechanisms underlying cuproptosis

### 3.1 Copper in cellular processes

#### 3.1.1 Copper homeostasis

Copper, an indispensable trace element, is crucial for a broad spectrum of physiological processes across nearly all cell types. Copper is integral to various biological functions, including iron metabolism, neurotransmitter synthesis, mitochondrial respiration, oxidative stress regulation, and energy production (122, 123). The current recommended daily intake of copper for healthy adults is 0.8–2.4 mg, while the total copper concentration in the human body ranges from 100 to 200 mg (124). To maintain systemic copper homeostasis within physiological limits, cells employ a complex regulatory network of copper enzymes, molecular chaperones, and membrane transport proteins, which collaboratively regulate copper uptake, efflux, and utilization (15, 24).

The absorption of dietary copper predominantly occurs in the small intestine and duodenum, primarily mediated by the copper transport protein 1 (CTR1), also known as solute carrier family 31 member 1 (SLC31A1), located on the apical side of intestinal epithelial cells (125). Dietary copper is mainly present in the form of divalent copper ions, upon transport to the cell surface in the blood, divalent copper ions are converted to monovalent copper ions by six-transmembrane epithelial antigen of the prostate (STEAP) proteins for further uptake (126). When intracellular copper levels are deficient, the expression of CTR1 is upregulated to facilitate the absorption of copper ions. However, under conditions of oxidative stress, the functionality of CTR1 may be compromised, thereby disrupting copper ion homeostasis (127). In addition, copper can also enter cells via divalent metal transporter 1 (DMT1) and through passive diffusion (128, 129).

Once inside the cell, copper ions are directly delivered to their target proteins by copper chaperones, which are a group of proteins responsible for transporting copper to specific intracellular sites and enzymes (130). The copper chaperone for superoxide dismutase (CCS) is specifically involved in the insertion of copper and the formation of disulfide bonds in Cu-Zn superoxide dismutase 1 (SOD1), and it regulates the localization of Cu-Zn SOD1 between the cytoplasm and mitochondria (131, 132). SOD1 is an enzyme ubiquitously present in cells, playing a crucial role in the elimination of superoxide radicals and protecting cells from oxidative damage; its mutations can lead to familial amyotrophic lateral sclerosis (ALS) (132, 133). The cytochrome c oxidase copper chaperone 17 (COX17), a copper metallochaperone, primarily responsible for transferring copper to synthesis of cytochrome oxidase 1/2 (SCO1/2) and cytochrome c oxidase copper chaperone 11 (COX11). SCO1/2 and COX11 are essential for the formation of cytochrome c oxidase 1 (COX1) and cytochrome c oxidase 2 (COX2) by delivering copper ions, respectively (122, 134). Notably, cytochrome c oxidase (CCO), the terminal complex in the electron transport chain (ETC) composed of two main components: COX1 and COX2, plays a crucial role in regulating intracellular biochemical processes (123, 135). Research indicates that the binding of copper to transcription factors via the antioxidant 1 copper chaperone (ATOX1) in the nucleus can further modulate gene expression (123). Additionally, ATOX1 facilitates the transport of copper to the trans-Golgi network proteins ATPase copper transporting alpha (ATP7A) and ATPase copper transporting beta (ATP7B). Both proteins function as P-type Cu-transporting ATPases, harnessing ATP hydrolysis energy to transfer copper ions across cellular membranes or to copper-dependent enzymes, thereby ensuring the maintenance of intracellular copper homeostasis (136, 137). Copper-transporting ATPases exhibit tissue-specific expression patterns. ATP7A is present in nearly all cells of the body, with the exception of hepatocytes. Copper is introduced into the bloodstream from enterocytes in the small intestine via the copper ion transporter ATP7A, which facilitates its systemic distribution (136, 138).

**Figure 2:**
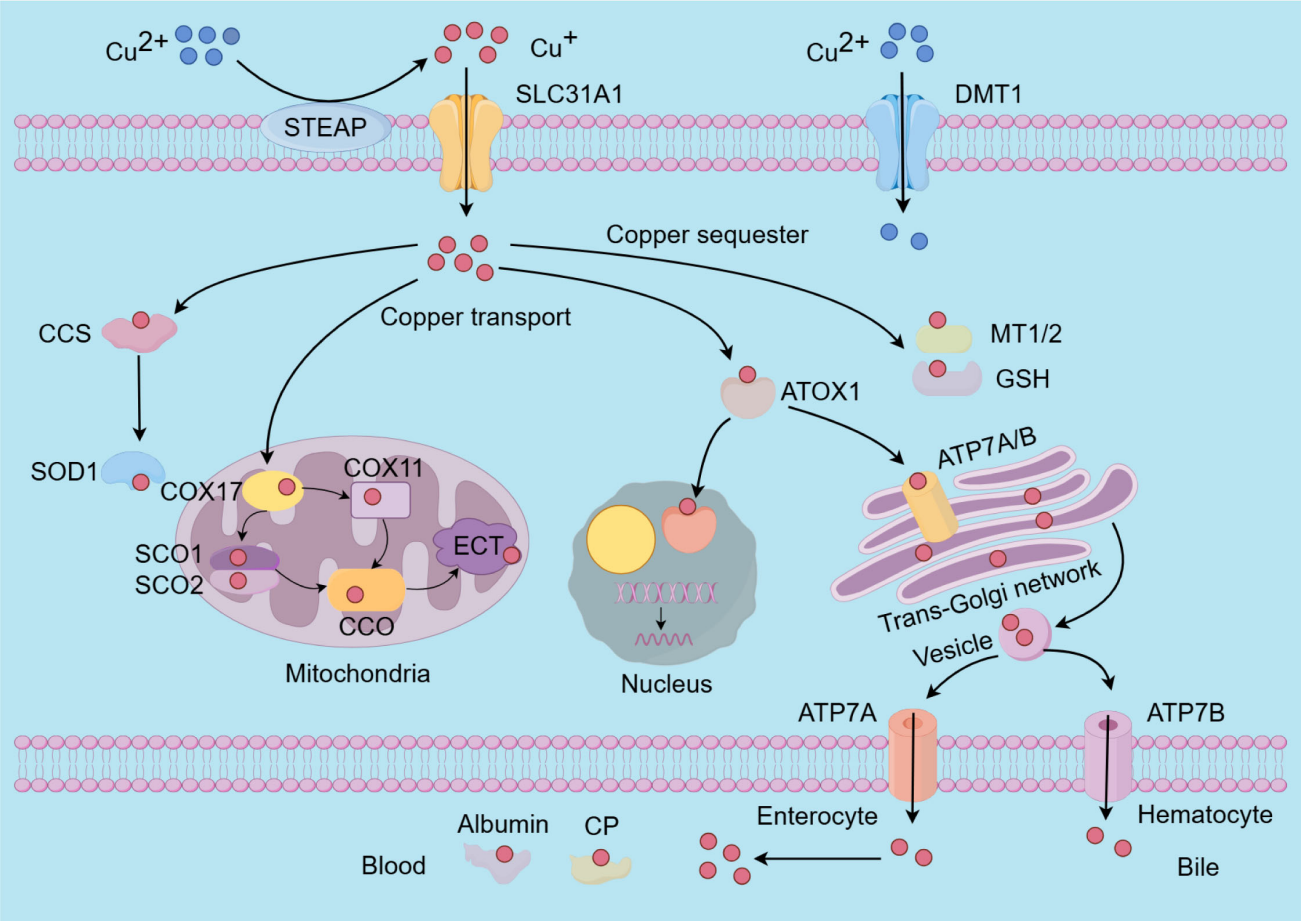
The molecular mechanisms underlying copper metabolism involve several key processes. The absorption of copper ions is primarily facilitated by the transporters SLC31A1 and DMT1. Notably, SLC31A1 is specific to the uptake of monovalent copper ions, necessitating the reduction of divalent copper ions to monovalent copper ions by STEAP before uptake. Within cells, copper is transported to and interacts with specific cytoplasmic copper chaperones, including COX17, CCS, and ATOX1, which direct copper to distinct cellular compartments such as the mitochondrial electron transport chain, the trans-Golgi network, and the nucleus. Copper insertion and disulfide bond formation in SOD1 are specifically facilitated by the CCS. To mitigate the cytotoxic effects of excess intracellular copper, copper ions can bind to MT1/2 and GSH for storage. Additionally, the maintenance of intracellular copper homeostasis is achieved through the export of surplus copper ions into the bloodstream via ATP7A and ATP7B. Subsequently, copper is transported throughout the body by CP and albumin. SLC31A1, Solute carrier family 31 member 1; DMT1, divalent metal transporter 1; STEAP, six-transmembrane epithelial antigen of the prostate; COX17/11, cytochrome c oxidase copper chaperone 17/11; CCS, cytoplasmic-mitochondrial metallochaperones; ATOX1, antioxidant protein 1; MT1/2, metallothioneins; GSH, glutathione; Cu-Zn SOD1, Cu-Zn superoxide dismutase 1; SCO1/2, sythesis of cytochrome oxidase 1/2; CCO, cytochrome oxidase; ATP7A/B, ATPase 7A/B; CP, cuproenzymes.

Copper predominantly attaches to ceruloplasmin (CP) and albumin in the bloodstream, and is then carried to the liver, which is the main site for copper storage and elimination. Surplus copper is expelled from liver cells into bile in vesicular form through ATP7B (139–141). Metallothioneins (MT1/2) and GSH serve as natural intracellular copper ion chelators, binding copper to store excess ions and prevent copper toxicity and cellular damage (142, 143) (**Figure 2**). The maintenance of intracellular copper homeostasis primarily depends on copper chaperones and transport proteins; an imbalance in copper homeostasis can result in cellular metabolic disorders and even cell death (144). This intricate equilibrium is essential for normal physiological processes and the prevention of disorders associated with copper dysregulation, including Alzheimer’s diseases (AD) and Parkinson’s diseases (PD) (145, 146).

In the CNS, ATP7A and ATP7B are crucial for regulating copper levels, with disruptions in this regulatory mechanism associated to MD and WD, respectively (147, 148). Severe copper deficiency can lead to impaired energy production due to mitochondrial cytochrome c oxidase dysfunction, while excessive intracellular copper levels may contribute to the onset of various diseases (149). Importantly, the redox properties of copper pose potential risks, as an excess can lead to the generation of harmful ROS, which may damage cellular components and contribute to the pathogenesis of cancer and CNS disorders (24, 131, 150).

### 3.2 Molecular components involved in cuproptosis

#### 3.2.1 Molecular mechanisms of cuproptosis

Although copper-induced cell death was recognized in the 1980s, the precise mechanism remained unclear (151). Over the past few decades, significant interest has emerged in exploring the relationship between copper and PCD, leading to extensive research on the mechanisms of copper-triggered cell death (8). The precise form of cell death induced by excessive copper ions has been a subject of debate, with researchers historically categorizing it as apoptosis, autophagy, or ferroptosis, all of which are closely associated with ROS and inflammatory responses (150).

It wasn’t until 2022 that the precise mechanism of copper-induced cell death was elucidated by Tsvetkov et al., who termed this process “cuproptosis” (6). This research challenges the traditional understanding of cuproptosis and initiates a new era of investigation into copper-induced cell death. Cuproptosis, a recently discovered type of cell death, is mediated by the ancient mechanism of protein lipoylation, a highly conserved post-translational modification that targets lysine residues (6, 150). This modification is exclusively observed in four specific enzymes: dihydrolipoamide S-succinyltransferase, glycine cleavage system H protein, dihydrolipoamide branched-chain transacylase E2 (DBT), and dihydrolipoamide S-acetyltransferase (DLAT). Notably, DLAT plays an essential role in the pyruvate dehydrogenase complex (PDC), facilitating the transformation of pyruvate into acetyl coenzyme A within the TCA cycle (123, 152).

The mechanism of cuproptosis involves a copper ionophore, elesclomol, which binds to divalent copper ions in the extracellular environment and facilitates their transport into cells. Within the mitochondria, elesclomol interacts with the direct target FDX1, subsequently releasing it. FDX1, a critical enzyme in the cuproptosis pathway, possesses a strong reducing capability, enabling it to lipoylate the aforementioned enzymes, and reduce divalent copper ions to monovalent copper ions (24, 153). Subsequently, monovalent copper ions directly interact with lipoylated mitochondrial protein fractions involved in the TCA cycle and affect Fe–S clusters, which are critical components of the ETC. This interaction induces the aggregation of lipoylated proteins, the loss of Fe-S cluster-containing proteins, and the upregulation of heat shock protein70 (HSP70). Consequently, the dysfunction of the TCA cycle and ETC occurs, ultimately leading to cell death mediated by proteotoxic stress (6, 153). Moreover, previous studies have demonstrated that HSP70 is significantly upregulated under conditions of stress, such as oxidative stress, hypoxia, and metal overload, thereby alleviating protein misfolding and aggregation to mitigate proteotoxic stress and prevent cellular damage (154, 155).

**Figure 3:**
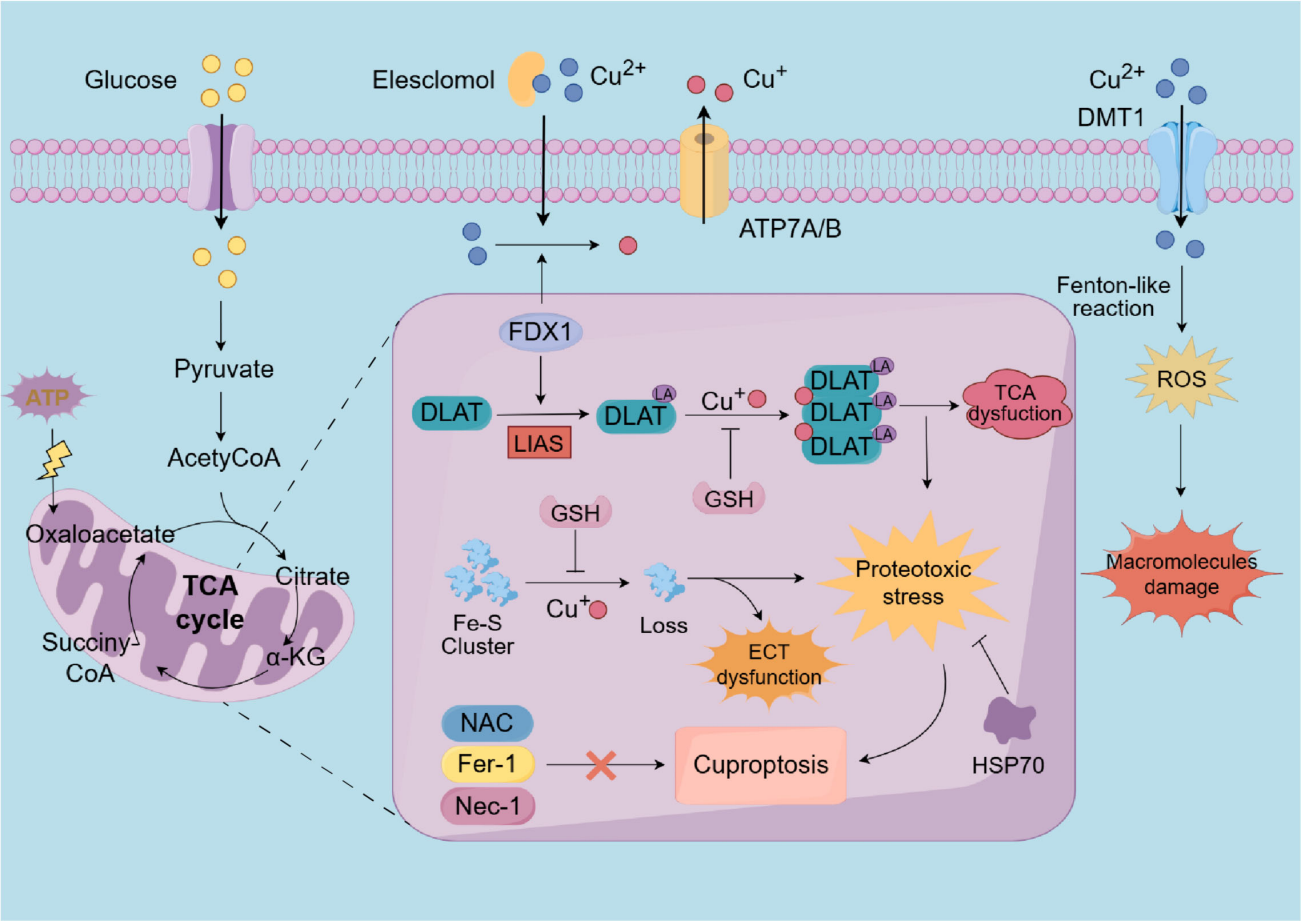
A diagrammatic representation of the cuproptosis process. Extracellular copper is sequestered by the copper ionophore elesclomol and transported into intracellular compartments. As an upstream regulator of protein lipoylation, FDX1/LIAS may enhance the lipoylation of TCA cycle enzymes, such as DLAT. Furthermore, within the mitochondria, FDX1 facilitates the reduction of divalent copper to its monovalent state. Subsequently, the monovalent copper could interact with DLAT and Fe-S cluster proteins results in DLAT aggregation and the depletion of Fe-S cluster proteins. These aberran interactions result in the TCA cycle and ETC to malfunction, triggering cuproptosis through proteotoxic stress, which eventually disrupts ATP synthesis. Notably, HSP70 and copper chelators, such as GSH, can inhibit cuproptosis. However, NAC, ferrostatin-1, and necrostatin-1 do not mitigate copper-induced cell death. Additionally, excessive copper ions can also induce oxidative stress and elevate ROS levels, thereby damaging intracellular macromolecules. FDX1, Ferredoxin 1; LIAS, lipoic acid synthetase; TCA, tricarboxylic acid; Fe-S, iron-sulfur cluster; DLAT, dihydrolipoamide S-acetyltransferase; ETC, electron transport chain; HSP70, heat shock protein 70; GSH, glutathione; NAC, N-acetylcysteine; ferrostatin-1, ferroptosis inhibitors; necrostatin-1, necroptosis inhibitors; ROS, reactive oxygen species.

In their study, Tsvetkov et al. have also demonstrated that the ROS inhibitor N-acetylcysteine (NAC), along with inhibitors targeting other established cell death pathways—such as ferroptosis inhibitors (ferrostatin-1), and necroptosis inhibitors (necrostatin-1)—were ineffective in mitigating cell death mediated by copper ionophores. Unprecedentedly, GSH, a cellular copper chelator, successfully prevented this process by binding with copper ions, highlighting the distinct nature of cuproptosis (6, 156) (**Figure 3**). Furthermore, the abrogation of cuproptosis was achieved through the knockout of seven genes, including FDX1 and six genes associated with the lipoic acid pathway (LIPT1, LIAS, and DLD) or protein targets of lipoylation (DLAT, PDHA1, and PDHB) (6). In contrast, the cuproptosis was up-regulated by inhibiting three genes, MTF1, GLS and CDKN2A (157, 158) (**Table 1**).

**Table 1:** Copper homeostasis and cuproptosis related regulatory proteins.

Protein	Type/Full name	Function	Ref.
SLC31A1	Cu transport protein	Absorption of copper ion	(125)
STEAP	Metalloreductase	Reduce divalent copper ions to monovalent copper ions	(126)
DMT1	Divalent metal ion transporter	Absorption of divalent copper ion	(128)
CCS	Copper chaperone	Regulate transport of copper and formation of disulfide bonds in SOD1	(131, 132)
SOD1	Cuproenzymes	Involving elimination of superoxide and prevention of oxidative damage	(133)
COX17	Copper metallochaperone	Responsible for mitochondrial copper transport	(122, 134)
SCO1	Mitochondrial copper chaperone	Delivering copper ions to cytochrome c oxidase 2 (COX2)	(122, 134)
SCO2	Mitochondrial copper chaperone	Delivering copper ions to cytochrome c oxidase 2 (COX2)	(122, 134)
COX11	Mitochondrial copper chaperone	Delivering copper ions to cytochrome c oxidase 1 (COX1)	(122, 134)
CCO	Cuproenzymes	Regulates intracellular biochemeical reactions	(123)
ATOX1	copper chaperone	Facilitates the transport of copper to organelles	(123, 137)
ATP7A	Copper efflux transporter	Facilitates copper ions systemic distribution	(136, 138)
ATP7B	Copper efflux transporter	Facilitates copper secrete from hepatocyte to bile	(140)
CP	Cuproenzymes	Facilitates copper ions systemic distribution	(141)
MT1/2	Metallothioneins	Chelate copper and prevent metal toxicity	(1)
GSH	Natural copper ion chelator	Chelate copper and prevent metal toxicity	(1)
FDX1	Ferredoxin 1	Reduce divalent copper ions to monovalent copper ions and lipoylate specific enzymes	(153)
DLAT	Dihydrolipoamide S-acetyltransferase	Lipoylated DALT oligomerization trigger proteotoxic stress and cell death	(153)
HSP70	Heat shock protein70	Reducing protein misfolding and aggregation to prevent proteotoxic stress	(154)
LIPT1	Lipoyltransferase 1	Involved in lipoic acid pathway and protein lipoylation	(159)
LIAS	Lipoic Acid Synthetase	Involved in lipoic acid pathway and protein lipoylation	(159)
DLD	Dihydrolipoamide Dehydrogenase	Involved in lipoic acid pathway and protein lipoylation	(159)
PDHA1	Pyruvate Dehydrogenase E1 Subunit Alpha 1	Positively regulates copper-triggered cell death	(8)
PDHB	Pyruvate Dehydrogenase E1 Subunit Beta	Positively regulates copper-triggered cell death	(8)
MTF1	Metal Regulatory Transcription Factor 1	Negatively regulates copper-induced cell death	(157)
GLS	Glutaminase	Negatively regulates copper-induced cell death	(158)
CDKN2A	Cylin Dependent Kinase Inhibitor 2A	Negatively regulates copper-induced cell death	(158)

#### 3.2.2 ROS generation and mitochondrial dysfunction

Every cell in the human body endeavors to maintain a balance between oxidant and antioxidant species, both of which are essential for cellular metabolism, signal transduction, and various biological functions (160). As an indispensable micronutrient, copper plays a crucial role in numerous biological processes to sustain cellular homeostasis. However, excessive copper disturbs the equilibrium between oxidants and antioxidants, resulting in ROS production and inducing oxidative stress, which then damages biological macromolecules like lipids, proteins, and DNA (24, 161). Additionally, elevated copper concentrations significantly impair the activity of Cu-Zn SOD1, catalase, and glutathione peroxidase, thereby compromising the antioxidant defense system and exacerbating the cytotoxic effects of ROS (162, 163). In patients with SCI, certain substances trigger reactions that damage the subcellular structures of neurons and glial cells, ultimately resulting in cell death. This phenomenon may be a primary factor contributing to the poor prognosis observed in these patients (12, 164).

Mitochondria, which are of great significance for cellular energy metabolism and maintaining mitochondrial homeostasis, also serve as the primary organelles responsible for generating ROS following SCI. Notably, ROS exacerbate mitochondrial damage, establishing a detrimental cycle that culminates in cell death (12). Copper-caused mitochondrial dysfunction and the promotion of mitochondrial fission are linked to the activation of mitophagy, which is closely associated with the upregulation of PINK1 and Parkin expression (165). Furthermore, copper has the potential to influence mitochondrial dynamics, including the processes of fission and fusion, which are vital for maintaining mitochondrial integrity and functionality, thereby contributing to cellular toxicity (166). Specifically, it has been suggested that the reduction of mitochondrial membrane potential is critical for copper-triggered astrocyte death, whereas neuronal damage is more closely associated with oxidative and nitrosative stress (167).

Historically, the specific mechanism of cell death induced by copper ions has been a subject of debate, and numerous studies have indicated a strong association with ROS (150). Evidence has demonstrated that copper directly targets complex IV (cytochrome c oxidase, CCO) of the ETC, upregulating its protein expression under copper overload conditions, without affecting other respiratory complexes (168, 169). Interestingly, Tsvetkov et al. identified a link between copper-induced cell death (cuproptosis) and mitochondrial metabolism, suggesting that copper impacts components of the TCA cycle rather than directly targeting the ETC (6).

In summary, while there is no direct correlation between ROS and the cuproptosis mechanism, ROS remain a critical factor in other pathways of copper-triggered cell death and should not be overlooked. The TCA cycle is intricately associated with cuproptosis due to its involvement in energy metabolism, redox homeostasis, and cellular signaling pathways. Further investigation is required to elucidate the specific mechanisms by which copper modulates TCA cycle dynamics and to understand how these interactions influence cell fate decisions in both physiological and pathological conditions.

#### 3.2.3 Ferredoxin1: the regulator of protein lipoylation and diseases

FDX1, an evolutionarily conserved protein characterized by the presence of Fe–S clusters, serves as an upstream regulator of protein lipoylation and is integral to the metabolism of steroids, cholesterol, and bile acids (170). FDX1’s involvement in cellular redox reactions and energy metabolism highlights its essential role in maintaining cellular homeostasis and its necessity for elesclomol-induced cuproptosis (24). Recent studies have emphasized the pivotal function of FDX1 across various cancer types, including clear cell renal cell carcinoma (ccRCC), hepatocellular carcinoma (HCC), and gliomas, where its expression levels have been associated with patient survival outcomes (171–173). In the molecular pathogenesis of HCC, increased FDX1 expression has been associated with enhanced immune cell infiltration, particularly involving natural killer cells and macrophages, potentially contributing to improved patient prognoses (172). Conversely, FDX1 deficiency may result in metabolic reprogramming and oxidative stress, which activate PINK1/Parkin-mediated mitophagy and the PI3K/AKT pathway, thereby facilitating HCC proliferation, invasion, and metastasis (174).

Furthermore, the expression of FDX1 is significantly diminished in aged ovaries compared to younger ones, indicating its potential as a biomarker and therapeutic target for age-related conditions (175). Modulating the expression of FDX1 may represent a promising strategy to enhance the efficacy of treatments designed to induce cuproptosis in cancer cells, potentially leading to novel therapeutic approaches in oncology (176). To summarize, FDX1 functions as a crucial regulator of cuproptosis, impacting cancer tumor progression, prognosis, immune infiltration, and therapeutic responses (174). Further investigation into the mechanisms and interactions of FDX1 could reveal new pathways for targeted therapies in both cancer and age-related diseases.

### 3.3 Cuproptosis in immune response

Copper is recognized for its immunomodulatory properties, affecting various components of the immune system. Recent research has underscored the significance of copper in modulating the innate immune response, especially through its interactions with immune cells and signaling pathways (177, 178). For example, copper has been demonstrated to augment the activity of alpha-kinase 1 (ALPK1), a cytosolic pattern-recognition receptor integral to host defense mechanisms against bacterial infections. This activation results in an elevated production of proinflammatory cytokines and an augmented recruitment of immune cells, thereby facilitating a robust immune response (179).

Additionally, over copper could induce cuproptosis—a newly explored PCD form mediated by elevated copper concentrations. Recently, numerous findings have shown that it is closely linked with the immune microenvironment in various tumors (178, 180). The regulation of cuproptosis in immune cells may impact their survival and functionality, thereby potentially influencing the overall immune response to pathogens and tumors (181). For instance, within acute myeloid leukemia mechanisms, specific patterns of cuproptosis regulation have been linked to varying immune profiles, indicating that modulating cuproptosis could serve as a strategy to enhance immunotherapy outcomes (182). The accumulation of copper at infection sites has been identified as a mechanism through which the host exerts antimicrobial effects, utilizing copper toxicity as a tool employed by phagocytic cell (183, 184). Copper plays a dual role as both an essential cofactor for immune function and a potential mediator of cell death, totally underscoring its intricate involvement in the immune response is vital for us. Consequently, the interaction between copper homeostasis, cuproptosis, and immune regulation represents a promising research avenue that may yield innovative therapeutic strategies for augmenting immune responses against infections and cancer.

### 3.4 Cuproptosis in disease states

#### 3.4.1 Cancer

The relationship between copper and tumorigenesis in cancer is complex, with dysregulated copper metabolism playing a dual role in both tumorigenesis and cancer therapy (185). Cuproptosis, a distinct form of cell death triggered by accumulated copper levels, has been shown to significantly impact the regulation of cancer cell survival and proliferation (8). This copper-dependent mechanism is unique compared to other types of PCD, such as apoptosis and ferroptosis, and involves specific TCA cycle pathways that can lead to proteotoxic stress under conditions of copper overload (6, 186). Interestingly, the tumor suppressor protein p53 may regulate the biogenesis of Fe-S clusters and GSH, both of which are critical components of the cuproptotic pathway, suggesting that p53 might play a significant role in mitigating copper toxicity and cuproptosis (187).

Emerging research has further elucidated the role of copper in cancer biology, highlighting its ability to promote tumor growth through cuproplasia—copper-dependent cellular proliferation—while also inducing cell death via the mechanism of cuproptosis (185). This complex interplay underscores the significance of copper metabolism as a pivotal factor in tumorigenesis, impacting both the initiation and progression of diverse cancer types (185). Copper ionophores, such as elesclomol and disulfiram, increase intracellular copper levels and induce cell death, which is mediated by oxidative stress, mitochondrial respiration disruption and protein lipoylation in cancer therapy (153, 188). Furthermore, the molecular mechanisms underlying cuproptosis remain under investigation, with current research efforts directed towards elucidating the interactions between this form of cell death and other cellular pathways and death mechanisms (186, 189). As the field of cancer research continues to evolve, the exploration of cuproptosis and its implications for tumorigenesis remains a promising area of investigation, with the potential to uncover novel insights into cancer biology and therapy (185, 190, 191).

#### 3.4.2 Metabolic disorders

In metabolic disorders, including diabetes, the regulation of copper homeostasis may be compromised, potentially resulting in aberrant cuproptosis within cells engaged in diverse pathophysiological processes (192). Copper is integral to enzymatic activity and cellular signaling. The dysregulation of copper levels can induce oxidative stress, mitochondrial dysfunction and even cuproptosis, a critical contributor to the pathogenesis of diabetic complications (161, 193).

Novel insights have underscored the significance of copper in the pathophysiology of diabetic retinopathy, indicating that disruptions in copper metabolism may play a role in vascular dysfunction and oxidative damage within retinal cells (194). Additionally, the newly defined concept of cuproptosis, closely associated with copper accumulation, implies that imbalances in copper homeostasis may adversely affect cellular viability and function in diabetic tissues (195, 196). Elucidating the mechanisms through which copper dysregulation impacts cuproptosis could yield valuable insights into prospective therapeutic strategies for addressing diabetes-related complications (193, 197). Specifically, interventions targeting copper metabolism, such as chelation therapy or the application of copper coordination complexes, may present novel treatment pathways (198).

These approaches have the potential to alleviate the detrimental effects of oxidative stress and enhance cellular health in individuals with diabetes (192, 196). In addition, the interaction between copper homeostasis and various metabolic pathways highlights the intricate nature of managing metabolic disorders. Ongoing research into the relationships among copper, cuproptosis, and metabolic health holds the potential to inform novel strategies aimed at improving treatment outcomes for diabetes and related conditions (196, 199, 200).

#### 3.4.3 Neurodegenerative diseases

In neurodegenerative disorders such as AD and PD, copper imbalance has been identified as a contributing factor (201, 202). The phenomenon of cuproptosis may be involved in the pathogenesis of these conditions, given that the homeostatic balance of copper within the brain is essential for sustaining neuronal health (202). Copper plays a critical role in numerous biological processes, including neurotransmission and antioxidant defense mechanisms. The dysregulation of copper levels can result in elevated oxidative stress, a major contributor to neurodegenerative processes (201, 203).

Copper ion imbalance has been observed in AD brains, where bivalent copper could attach to amyloid β (Aβ) protein, leading to the production of ROS and subsequent neuronal damage (204, 205). Copper consumption and environmental copper levels are significant risk factors for PD, whereas the copper chelator ATH434 therapy lowers copper levels to ease motor and olfactory issues in PD patients (202, 206). Moreover, numerous studies have demonstrated that in AD, copper dyshomeostasis is correlated with the accumulation of Aβ plaques and neurofibrillary tangles, which are hallmark features of the disorder (145, 207). In a similar vein, in PD, dysregulated copper levels have been associated with the aggregation of α-synuclein, a protein that constitutes Lewy bodies within affected neurons (146). Furthermore, the interaction between copper and other metals, such as iron and zinc, further complicates the neurodegenerative milieu, as imbalances can intensify oxidative damage and neuronal death (208, 209).

Notably, within the context of SCI, the BSCB integrity is compromised, leading to an immediate increase in its permeability, allowing a large influx of copper ions in the bloodstream pour into the spinal cord tissue and causing a copper overload situation (210, 211). Subsequently, the oxidative stress, inflammatory, and immune responses induced by SCI further disrupt the normal functioning of intracellular copper ion transport proteins, result in an imbalance in copper homeostasis and possibly initiating cuproptosis (131, 211). Nevertheless, the precise moments for the accumulation of copper ions and the onset of cuproptosis post-injury are not yet determined.

Recent research has underscored the potential of targeting copper-related pathways for therapeutic interventions. Specifically, copper-modulating therapies are currently under investigation in clinical trials for AD, with the anticipation that these therapies may also prove beneficial for patients with PD (212, 213). Collectively, the dysregulation of copper within the CNS constitutes a pivotal factor in the onset and advancement of neurodegenerative disorders and SCI. Additional research is imperative to elucidate the specific mechanisms through which copper modulates relevant processes and to investigate potential therapeutic strategies aimed at targeting copper homeostasis and cuproptosis (202, 213).

## 4 Role of cuproptosis in spinal cord injury

### 4.1 Cuproptosis and other programmed cell death in SCI

**Figure 4:**
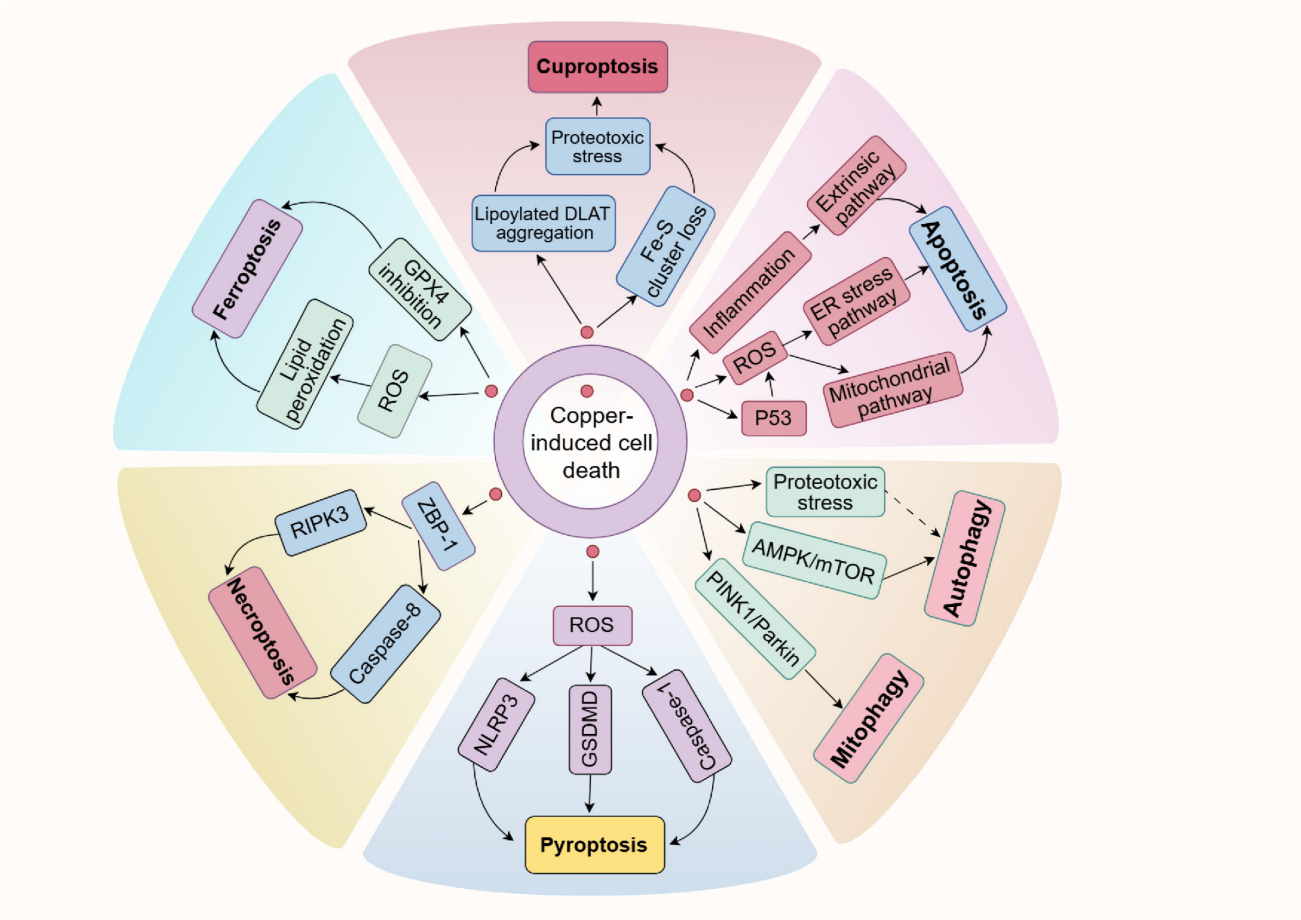
Copper is closely associated with various forms of PCD. On the one hand, an excess of copper can enhance p53 expression and ROS levels or initiate inflammation, causing cell apoptosis. On the other hand, copper exposure increases ROS levels or reduces GPX4 expression, which subsequently leads to ferroptosis or pyroptosis via specific pathways. It has been shown that copper induces necroptosis mediated by ZBP1. Furthermore, copper can also trigger autophagy and mitophagy by inhibiting the AMPK/mTOR pathway or activating the PINK1/Parkin signaling pathway, respectively. It is noteworthy that recent research has shown copper could induce cuproptosis, a newly discovered cell death type, characterized by the accumulation of lipoylated proteins and the depletion of Fe-S clusters. Possible connections are shown with dashed arrows.

While copper ionophores interacting with copper can induce cuproptosis, previous studies have also found that excessive copper in cells could trigger other cell death pathways, including apoptosis, autophagy, pyroptosis, necroptosis and ferroptosis (**Figure 4**). This suggests a notable interaction of molecular mechanisms between cuproptosis and other forms of cell death. Moreover, we further elaborated on and compared the key differences in mechanisms for these PCD (**Table 2**). Comprehensively understanding the role of copper in other cell death pathways may help develop more effective SCI treatment strategies targeting cuproptosis.

**Table 2:** The differences of cuproptosis and other PCD.

Type	Core mechanisms	Major triggers	Major inhibitors	Ref.
Cuproptosis	Lipoylated proteins aggregation and Fe-S cluster loss	Copper overload and mitochondrial proteotoxic stress	Rotenone, antimycin A, D-penicilamine, and GSH	(8, 24, 214)
Apoptosis	The activation of endogenous caspases	Oxidative stress, inflammation, and excitotoxicity	Q-VD-OPh and zVAD-fmk	(68, 215)
Autophagy	The formation of phagophores, autophagosomes, and autolysosomes	Oxidative stress, nutrient deprivation, and mechanical injury	Bafilomycin A_1_, 3‐methyladenine (3-MA), and chloroquine (CQ)	(216, 217)
Pyroptosis	NLRP3/AIM2 inflammasome and caspase-4/5/11 pathways	Infection, immune activation, and mechanical trauma	MCC950, betulinic acid (BA), and topotecan (TPT)	(96, 97, 100)
Necroptosis	RIPK1/3 and MLKL pathways	Inflammatory and immune response	Necrostatins, KW-2449, and piperlongumine	(108, 218, 219)
Ferroptosis	GPX4-dependent and ACSL4-dependent pathways	Iron overload and lipid peroxidation	Ferrostatin-1, celastrol, and deferoxamine (DFO)	(117, 118, 121)

#### 4.1.1 Link with apoptosis

The crosstalk between cuproptosis and apoptosis constitutes an emerging area of research, particularly in the pathomechanistic continuum of SCI. Previous studies have demonstrated that excessive copper exposure can induce oxidative stress through the overproduction of ROS, subsequently promoting hepatic apoptosis via the mitochondrial pathway or ER stress pathway (64, 220, 221). Specifically, in MCF7 human breast cancer cells, copper ions have been observed to elevate the expression of p53, subsequently inducing the production of ROS and cell apoptosis (222). In this case, p53 may mitigate cuproptosis by regulating the expression of crucial signaling molecules, including Fe-S clusters and the copper chelator GSH (187).

Additionally, excess copper is also implicated in various inflammatory vascular diseases, whereas copper chelators have been shown to inhibit inflammation (223). Inflammation is characterized by the secretion of various pro-inflammatory cytokines, which initiate the extrinsic pathway of apoptosis following SCI (45, 70). Nuclear factor kappa B (NF-κB) is a key activator of inflammatory processes; inhibiting its expression may thus be a promising strategy for treating SCI and other inflammatory diseases (99, 131, 224).

These studies indicate that ROS, p53, and inflammation may mediate the crosstalk between cuproptosis and apoptosis. Within the process of SCI, the pathological milieu is marked by oxidative stress and inflammation, both of which can intensify neuronal damage and impede recovery. By elucidating the mechanisms of this crosstalk, researchers may uncover novel therapeutic targets to enhance recovery and improve the quality of life for individuals affected by SCI.

#### 4.1.2 Link with autophagy

Copper, recognized for its involvement in numerous biological processes, can induce oxidative stress and the ROS levels, potentially eliciting autophagic responses (225, 226). In SCI pathology, autophagy exhibits dual roles, functioning as both protective and detrimental processes. When overactivated, the autophagic machinery can enhance cell death by selectively degrading proteins that protect against damage or oxidative stress (227). However, some studies have shown that autophagy is an essential cytoprotective pathway and blocking autophagy in neurons and oligodendrocytes will limit functional recovery after SCI (74, 228, 229). Furthermore, the interaction between copper concentrations and autophagy may hold substantial significance in the progression of diseases such as neurodegeneration, wherein both cuproptosis and autophagy are integral to cellular survival and function (24, 202, 230). As mentioned above, excessive copper could lead to the TCA and ECT dysfunction, ultimately inducing cellular death mediated by proteotoxic stress (6, 153). Meanwhile, this process may induce autophagy respone to clear or recycle misfolded proteins, thereby mitigating cellular toxicity (72).

The regulation of autophagy in relation to copper is complex and involves multiple signaling pathways. Research has demonstrated that exposure to copper results in liver metabolic issues and suppresses the AMPK/mTOR signaling pathway, which in turn triggers liver autophagy (231). Moreover, copper exposure can induce mitophagy via the PINK1/Parkin pathway in chicken livers, potentially mitigating copper-induced mitochondrial apoptosis (232). Collectively, these studies enhance our understanding of copper-triggered cell death, suggesting that the initiation of cuproptosis may also influence autophagic activity.

#### 4.1.3 Link with pyroptosis

Copper exposure has been implicated in the induction of NLRP3-dependent cellular pyroptosis, which serves as a mediator of inflammation and neurological toxicity (233). In chicken hepatocytes, copper-induced ROS have been shown to activate the NLRP3 inflammasome and promote the expression of caspase-1 protein, subsequently leading to pyroptosis. The application of NAC, a ROS scavenger, and Z-YVAD-FMK, a caspase inhibitor, has been found to mitigate the excessive pyroptosis induced by copper (234). This study suggests that ROS generated by copper accumulation may play a critical role as mediators of pyroptosis. Similarly, Zhou et al. demonstrated that copper exposure elevates oxidative stress and ROS levels in MN9D cells, which subsequently result in the upregulation of GSDMD protein and inflammasome-mediated pyroptosis (235). Additionally, copper ions can interfere with mitochondrial copper balance, leading to Aβ aggregation and increased levels of NLRP3, caspase-1, and GSDMD, which in turn activate pyroptosis in AD cell models (236). The prospective involvement of copper disturbance and cuproptosis in this context introduces novel research opportunities aimed at devising effective therapeutic strategies for managing SCI-associated cell death and facilitating recovery.

#### 4.1.4 Link with necroptosis

Exposure to nano-copper has been shown to induce Z-DNA combined protein-1 (ZBP-1)-mediated necroptosis in the immune organs of chickens, while hesperidin appears to mitigate the toxic effects associated with nano-copper exposure (237). Jiao and colleagues have demonstrated that the activation of ZBP1 results in the recruitment of RIPK3 and caspase-8, which subsequently activate the NLRP3 inflammasome, thereby triggering necroptosis (238). A bioinformatics analysis further suggests a link between ZBP1 and both cuproptosis and necroptosis (239). Moreover, a recent study indicates that copper can activate autophagy via ULK1 and ATG16L1, which in turn may attenuate RIPK3- and MLKL-mediated necroptosis (240). Although there is currently no evidence addressing the interaction between cuproptosis and necroptosis in patients with SCI, these findings imply a potential correlation and crosstalk between copper-exposure-induced cell death and necroptosis signaling pathways.

#### 4.1.5 Link with ferroptosis

Cuproptosis and ferroptosis are metal-associated forms of cell death, characterized by both similarities and differences in their underlying mechanisms, as previously discussed. While earlier research suggested that ferroptosis is exclusively triggered by metal ions, emerging evidence indicates that copper can also promote ferroptosis under certain conditions (24, 241). Gai and colleagues have demonstrated that copper can initiate the ferroptosis process by accumulating lipid peroxides and inhibiting the activity of GPX4 (242). GSH, an essential cofactor for GPX4, directly influences the enzyme’s activity and stability, leading to lipid peroxidation and ferroptosis in cells (113). The accumulation of copper or iron within cells results in increased GSH consumption, thereby initiating cuproptosis and creating a favorable environment for ferroptosis (226). Intriguingly, buthionine sulfoximine (BSO), known as a ferroptosis inhibitor due to its role in reducing GSH synthesis, has also been observed to induce cuproptosis (6).

In the oxidative phosphorylation process, iron plays a critical role in mitochondrial ATP synthase, with numerous Fe-S clusters present in Complex I and Complex II (243). Recent studies has indicated that the depletion of Fe-S cluster proteins can lead to ferroptosis, while the reduction of Fe-S clusters is also a key mechanism in cuproptosis (244). The TCA cycle is fundamental to cellular metabolism, not only facilitating the oxidation of nutrients to generate energy and providing essential metabolites for biosynthetic processes, but also being intricately linked to the ETC in ATP production (245). Blocking the mitochondrial TCA cycle or ETC can reduce cystine-deprivation or erastin-induced ferroptosis, suggesting that the TCA cycle is crucial for ferroptosis (246). Furthermore, the TCA cycle is crucial for cuproptosis, with the aggregation of lipoylated DLAT and the loss of Fe-S clusters proteins being a primary inducer of copper-induced cell death (6). These findings imply that there may be an interaction between cuproptosis and ferroptosis through the TCA cycle and ETC.

An analysis of genomic data across 33 cancer types reveals intricate molecular interactions between the regulators of cuproptosis and ferroptosis at the multiomic level (247). Additionally, both iron and copper have the capacity to elevate ROS levels by promoting GSH depletion or facilitating the Fenton reaction, which is pivotal in elucidating the interplay between ferroptosis and cuproptosis (226). Although cuproptosis is primarily attributed to the aggregation of lipoylated proteins in the TCA cycle rather than ROS generation, it continues to play a crucial role in other forms of copper-triggered cell death, such as ferroptosis and apoptosis (6, 222, 226, 248). To conclude, while cuproptosis and ferroptosis have unique molecular mechanisms and traits, they might influence each other through critical signaling pathways and interactions of protein molecules within the context of SCI.

### 4.2 Impact of cuproptosis on the spinal cord microenvironment after injury

#### 4.2.1 Inflammatory response

Following SCI, an immediate inflammatory response is initiated, which significantly influences subsequent pathophysiological processes. This response is marked by the recruitment of immune cells, including neutrophils, monocytes, and macrophages, to the site of injury, which plays a crucial role in wound healing and tissue regeneration (40, 249). However, excessive inflammation can exacerbate tissue damage and hinder recovery, thereby contributing to secondary injury (23). Current studies have elucidated the involvement of various cellular mechanisms in the regulation of the inflammatory response. Notably, the chemokine CCL2 has been shown to significantly impact inflammation through the PI3K/Akt signaling pathway, suggesting that interventions targeting this pathway may offer therapeutic benefits in managing inflammation following SCI (250). Additionally, microRNAs, such as miR-182, have been identified as critical regulators of inflammation and apoptosis in neurons post-SCI, indicating that their modulation could enhance recovery outcomes (251).

Li et al. suggest that copper overload may cause heightened polarization of peripheral M2 macrophages and significantly decrease T cell numbers, with cuproptosis possibly affecting inflammatory pathway activation and worsening the immune microenvironment within the injured spinal cord, thereby impacting prognosis (10). The interaction between cuproptosis and traditional inflammatory responses warrants further investigation, as it may uncover novel therapeutic targets for mitigating inflammation and enhancing recovery post-SCI. Moreover, copper exposure could induce the dysfunction of multiple inflammatory-related pathways, such as NF-κB signaling pathway, a crucial regulator of inflammation in SCI, and the inhibition of NF-κB pathway has been associated with improved clinical outcomes (251–253). Investigating the interaction between cuproptosis and these well-established pathways may offer valuable insights into novel strategies for managing inflammation and promoting recovery in patients with SCI.

#### 4.2.2 Glial cell response

Glial cells, encompassing microglia, astrocytes and oligodendrocytes, are integral to the functioning of the spinal cord. Microglia play a critical role in the development of the glial scar post-SCI, and the reduction of microglia disrupts glial scar formation, subsequently increasing parenchymal immune infiltrates, decreasing neuronal and oligodendrocyte survival, and adversely effecting on motor function and prognosis (254). Astrocytes, which are pivotal for maintaining the BSCB and providing neurotrophic support, can undergo reactive gliosis following injury. Although this process initially serves a protective function, it can result in the formation of a glial scar that may ultimately impede axonal regeneration and contribute to secondary damage (255, 256). Studies have shown that astrocytes and microglia are crucial for maintaining copper metabolism. Disruptions in this balance, such as copper accumulation or disease states, can impair neuronal health and promote neurodegenerative processes (230). Furthermore, oligodendrocytes are tasked with the myelination of axons within the CNS. Following SCI, oligodendrocyte progenitor cells (OPCs) undergo proliferation and differentiation to facilitate the remyelination of damaged axons, a process critical for the restoration of neuronal function (257).

Mao and colleagues have shown that Mpeg1 is significantly expressed in microglia after SCI. It may decrease cuproptosis, lessen the inflammatory response during SCI, and potentially protect spinal cord tissue from harm (22). Moreover, the interactions among astrocytes, oligodendrocytes, and microglia are pivotal in the context of immunity. In patients with SCI, DLD, a regulator of cuproptosis, can positively regulate cuproptosis, induce the polarization of M2 macrophages, and ultimately affect the immune microenvironment and prognosis (10).

Although there is scarce experimental evidence directly linking copper overexposure to cuproptosis in neural cells, numerous studies in other cell types and tissues underscore the crucial role of cuproptosis as a mechanism of copper-induced cytotoxicity. Regrettably, it remains unclear whether cuproptosis specifically impacts spinal cord neurons, glial cells, or immune cells due to a lack of cell-type-specific studies after SCI. The dysregulation of these glial responses, due to copper accumulation and cuproptosis, may impair their ability to support neuronal survival and repair, thereby exacerbating neuronal loss and functional impairments (201, 230). Elucidating the mechanisms through which cuproptosis influences the function of neurons, glial cells, and immune cells in SCI is pivotal for the development of targeted therapeutic strategies that enhance neuroprotection and facilitate recovery.

### 4.3 Therapeutic implications

#### 4.3.1 Modulating cuproptosis as a therapeutic strategy

Given the involvement of cuproptosis in SCI, modulating this process may represent a promising therapeutic strategy. This could involve either inhibiting cuproptosis to prevent excessive cell death or promoting it to eliminate damaged cells that contribute to the pathology of the injury. Recent research highlights the significance of cuproptosis in various neurological conditions, such as stroke, cancer, and neurodegenerative diseases, suggesting that understanding its mechanisms could aid in developing novel treatment strategies for SCI (185, 258, 259).

Within the pathomechanical cascade of SCI, the dysregulation of copper metabolism and cuproptosis may exacerbate neuronal damage and hinder recovery processes. A recent investigation identified that several crucial cuproptosis-related genes (CRGs), such as ATP7A, CP, copper-dependent lysyl oxidase-like 2 (Loxl2), and Phosphodiesterase 3B (PDE3B), exhibit upregulation in neurons following SCI, thereby initiating cuproptosis and neuroinflammation at the central lesion sites. Interestingly, treatment with neurotrophin-3 (NT3)-loaded chitosan markedly suppressed the expression of these genes and facilitated functional recovery in animal models of SCI (260). In addition, among individuals with SCI who do not receive timely intervention, serum copper levels increase within 24 hours, indicating a strong correlation with clinical outcomes (11). Zhou et al. revealed that seven CRGs exhibited distinct expression patterns in SCI patients, with ATP7B, DLD, and SLC31A1 showing increased expression, while DLST, DBT, LIAS, and LIPT1 showed decreased expression (211). These alterations might affect mitochondrial function, oxidative stress, and immune response, potentially hindering the recovery of neurological function in SCI patients. Therefore, targeting cuproptosis offers a potential strategy to restore copper homeostasis and mitigate the detrimental effects of copper overload on neuronal and glial cells. This approach aligns with emerging research emphasizing the importance of cellular and molecular therapies in augmenting neuroprotection and facilitating functional recovery following SCI (10, 22).

Moreover, the investigation into copper ionophores and nanoparticle delivery systems, which have demonstrated potential in cancer therapy, may be repurposed for the treatment of SCI. These approaches may enable the precise modulation of cuproptosis, thereby improving therapeutic efficacy while minimizing potential adverse effects (24, 202). Beyond this, emerging evidence suggests that p53, HSP70 and autophagy-related proteins may cooperatively mitigate cuproptosis by enhancing Fe-S cluster biogenesis, alleviating associated proteotoxic stress, respectively, thereby restoring mitochondrial function. As research advances, it will be crucial to clarify the exact pathways of cuproptosis and how they interact with other cell death processes like apoptosis, autophagy, and ferroptosis to develop comprehensive treatment protocols for SCI (226, 261). To summarize, the modulation of cuproptosis represents a promising new approach for therapeutic intervention in SCI, necessitating further exploration of its underlying mechanisms and potential clinical applications.

#### 4.3.2 Copper - chelating agents

Originally developed for the treatment of WD, copper chelators have recently been investigated as inhibitors of cuproptosis in recent years (24, 262). These agents can decrease the bioavailability of copper, potentially reducing oxidative stress and cellular toxicity linked to high copper levels, which are involved in numerous pathological processes such as neurodegeneration and cell death (8, 263).

Tetrathiomolybdate (TTM) is one of the earliest drugs developed for the treatment of WD, primarily by modulating copper homeostasis (264). Furthermore, research by Kim et al. has demonstrated that in ovarian and endometrial tumors, TTM can downregulate HIF-1α through a mechanism dependent on HIF-prolyl hydroxylase and inhibit the function of the copper-dependent mitochondrial complex IV (265). D-penicillamine, another prominent copper chelator used in the treatment of WD, could also be used as a supplementary treatment in conventional cancer therapies. The potential mechanism involves metal ion-catalyzed H_2_O_2_-mediated oxidative stress, which selectively induces cancer cell death and enhances radio- and chemo-sensitization (8, 266). Interestingly, GSH, a natural copper ion chelator, can directly bind to excess intracellular copper, thereby reducing proteotoxic stress and preventing cellular cuproptosis (6).

During SCI progression, the application of copper chelators and specific proteins may restore copper homeostasis, thereby alleviating the detrimental effects associated with copper overload. However, there is no evidence showing that these chelators are applied after SCI at present. Additionally, the potential synergistic effects of combining copper chelators with antioxidants or anti-inflammatory drugs are still speculative and require further experimental validation. Future researches should comprehensively elucidating the molecular mechanisms underlying cuproptosis and utilize specific inhibitors that against it, which might offer valuable insights for the development of targeted therapies for SCI, with the potential to enhance recovery outcomes and mitigate secondary damage.

The interaction between copper metabolism and cellular processes underscores the critical importance of sustaining copper homeostasis and cuproptosis, particularly in conditions such as SCI, where oxidative stress, inflammatory responses and mitochondrial dysfunction are prominent. Future research should aim to elucidate the precise pathways implicated in copper-triggered cell death and explore the potential of chelation therapy to modulate multiple pathways, thereby providing novel therapeutic strategies for neurotrauma.

## 5 Conclusion

Cuproptosis represents a newly identified type of cell death characterized by distinct pathophysiological mechanisms. Although significant progress has been made in comprehending the intricacies of cuproptosis in recent years, numerous aspects of this process remain incompletely understood. Current research actively explores the role of copper in cellular processes, the molecular components involved in cuproptosis, and the implications of this form of cell death in numerous pathological states. Further research into cuproptosis will not only deepen our comprehension of fundamental biological processes but also hold significant implications for the development of novel therapeutic strategies for a range of diseases. Additionally, cuproptosis may exhibit a complex and multifaceted role in SCI. Elucidating its molecular mechanisms, its interactions with other cell death pathways, its effects on the spinal cord microenvironment, and its therapeutic potential is essential for the advancement of effective treatments for SCI. Future research should aim to further elucidate these aspects of cuproptosis in SCI, with the ultimate objective of improving prognosis and quality of life for individuals affected by SCI.
